# Cell Differentiation Trajectory Predicts Prognosis and Immunotherapeutic Response in Clear Cell Renal Cell Carcinoma

**DOI:** 10.1155/2022/8422339

**Published:** 2022-11-29

**Authors:** Jin Xu, Xi Chen, Yinyu Chen, Qiushuang Wang, Yingliang Jin, Huashuo Zhao

**Affiliations:** ^1^Department of Biostatistics, School of Public Health, Xuzhou Medical University, Xuzhou, Jiangsu 221004, China; ^2^School of Stomatology, Xuzhou Medial University, Xuzhou, Jiangsu 221004, China

## Abstract

Clear cell renal cell carcinoma (ccRCC) is the main type of malignancy in kidney related to glucose metabolism. Primary single cell culture and single cell sequencing are novel research technologies. In this study, we explored the differentiation status of ccRCC cells and its significance in prognosis and immunotherapeutic response through bioinformatics. We characterized distinct differentiation states and differentiation-related genes (DRGs) in ccRCC cells through single cell RNA sequencing (scRNA-seq) analysis. Combined with bulk RNA-seq data, we classified patients into two clusters and found that this classification was closely correlated with patient prognosis and immunotherapeutic responses. Based on machine learning, we identified a prognostic risk model composed of 14 DRGs, including BTG2, CDKN1A, COL6A1, CPM, CYB5D2, FOSB, ID2, ISG15, PLCG2, SECISBP2, SOCS3, TES, ZBTB16, and ZNF704, to predict the survival rate of patients and then constructed a nomogram model integrating clinicopathological characteristics and risk score for clinical practice. In the study of immune checkpoints, we found that patients in the high-risk group had a disposition to get worse prognosis and better effects of immune checkpoint blocking therapies. Finally, we found the expression level of model DRGs was associated with a tumor-immune microenvironment (TIME) pattern and the response of 83 compounds or inhibitors was significantly different in the two risk groups. In a word, our study highlights the potential contribution of cell differentiation in prognosis judgment and immunotherapy response and offers promising therapeutic options for ccRCC patients.

## 1. Introduction

Clear cell renal cell carcinoma (ccRCC) is the main type of RCC, accounting for about 70% of adult clinical cases. ccRCC is characterized by the loss of von Hippel-Lindau and the accumulation of robust lipid and glycogen [1]. Local ccRCC can be detected early and successfully treated by surgery, while metastatic ccRCC is almost always fatal. The lack of sensitivity to chemotherapy and radiation therapy has brought great trouble to clinicians and also brought huge burden to patients. Over the past decade, several targeted agents and immunotherapies have been added to the treatment plan of metastatic ccRCC [2–4]. Due to the high heterogeneity of ccRCC, previous classifications cannot satisfactorily predict the prognosis of patients with the same diagnosis [5, 6]. Furthermore, although countless prognostic models with significant genes have been constructed, the accuracy of their prediction performance still needs to be further confirmed and improved in clinical practice [7–9]. Immunotherapy, as a new therapy, has been widely used in multiple tumors [10]. Clinical practice has proved that immunotherapy had a good effect on most cancers and thus immunotherapy for ccRCC has been paid increasing attention by researchers [11, 12]. However, due to the lack of accurate predictive biomarker selection, only a few ccRCC patients have achieved an effective immune response in clinical trials [13, 14]. Therefore, it is urgent to construct an effective classification or biological prediction index to distinguish the prognosis and immunotherapy response of ccRCC patients.

Compared with the traditional bulk RNA sequencing technology, which can only reflect the average variation level of tumor for all cells in the sample, the single-cell sequencing (scRNA-seq) technology has provided unprecedented molecular resolution for researchers to study cancer [15, 16]. Tumor characteristics hidden in the heterogeneity of cell population could be revealed through single-cell genomics and trajectory analysis, which could offer possible prognostic biomarkers for better individualized treatment [17, 18]. However, the clinical samples of scRNA-seq research are limited and cannot be associated with clinicopathological data. In this case, the utilization of scRNA-seq data could be optimized by integrating bulk RNA-seq data. So far, few studies have focused on the construction of prognostic risk based with differentiation-related genes (DRGs). It is also unclear whether the novel classification based on cell differentiation trajectory is related to tumor biological behavior and whether cell differentiation plays a part in predicting the prognosis and immunotherapeutic response of ccRCC patients.

In this study, transcriptomic data of ccRCC samples were used to test our deduction. First of all, we used scRNA-seq data to identify ccRCC cell subsets in diverse differentiation states and significant DRGs through trajectory analysis. Second, we employed bulk RNA-seq data to classify ccRCC patients based on these DRGs and proved that this novel classification showed significantly different prognoses and immunotherapy responses. Third, 14 DRGs were identified as key genes-related ccRCC prognosis and a prognostic risk model was constructed by these DRGs. Next, we comprehensively made an exploration of TIME and drug sensitivity based on this 14-DRGs prognostic risk model. At last, a clinically applicable nomogram integrating clinicopathological characteristics and risk score was developed successfully for ccRCC patients.

## 2. Methods

### 2.1. Data Acquisition

The scRNA-seq data and bulk RNA-seq data of ccRCC samples included in this study are available in the Cancer Genome Atlas (TCGA, https://portal.gdc.cancer.gov/) and the Gene Expression Omnibus (GEO, https://www.ncbi.nlm.nih.gov/geo/) database [19, 20]. Bulk RNA-seq data and available clinical information were available for 519 samples in the TCGA-KIRC dataset and 39 samples in the GSE29609 dataset [21]. The corresponding clinicopathological characteristics are listed in the Supplementary Tables 1 and 2. In addition, the scRNA-seq data were obtained from the GSE156632 dataset and contained 35,433 cells from tumor tissues of 7 ccRCC patients. Supplementary Table 3 presents the corresponding clinicopathological features.

### 2.2. Processing of the scRNA-seq Data

The “Seurat” package was employed for the scRNA-seq data processing, including quality control, data exploration, statistical analysis, and result visualization [22]. First, low quality cells were excluded according to the following quality control criteria: (1) genes detected in <500 cells; (2) cells with <1,000 or >20,000 detected genes; and (3) cells with >10% of mitochondrial expressed genes. Then, the batch effects of the scRNA-seq data were corrected by “harmony” package [23]. Third, the scRNA-seq data were normalized through “LogNormalize” method and subsequently, top 1,000 highly variable genes were identified by “VST” package [24]. Next, principal component analysis (PCA) was used for dimensionality reduction of ccRCC cells to determine the significantly available dimensions (*P* < 0.05) [25]. Based on top 11 PCs, the uniform manifold approximation and projection (UMAP) algorithm was utilized for dimensionality reduction and clustering across all ccRCC cells [26]. Genes with the cutoff criteria of adjusted *P* < 0.05 and |log_2_ fold change (FC)| > 1 were regarded as the marker genes in each cluster through “limma” package [27]. Finally, according to the marker genes, these clusters were annotated using “singleR” package and manually verified and corrected through the CellMarker database and references [28, 29].

### 2.3. Trajectory Analysis and DRGs Identification

Trajectory analysis could reduce high-dimensional representations to low-dimensional space by reducing master map learning. Cells were casted into this space and arranged as a trajectory with branch points. In addition, cells distributed in the same branch were considered to have similar differentiation status and characteristics. These genes with different expression levels among branches were identified and defined as differentiation-related genes (DRGs). Therefore, this study analyzed the trajectories of renal tubular and cancer cells by the “Monocle 2” package [30] and the enrichment analysis for these DRGs in different branches was performed using “clusterProfiler” package [31].

### 2.4. Classification for ccRCC Patients According to DRGs

The data of the TCGA-KIRC dataset were performed to make a transformation to transcripts per million (TPM) values and merged with GSE29609 dataset as training cohort [32]. The training cohort was normalized with log_2_ scale transformation and the batch effect was corrected by “SVA” package [33]. These processed expressions of DRGs were subsequently extracted for nonnegative matrix factorization (NMF). Then, “survival” package was employed for the cox regression analysis to explore the correlation between the expression patterns of all DRGs and overall survival. DRGs with *P* < 0.01 were considered to be significantly associated with prognosis and selected for further analysis. Next, the unsupervised clustering method of NMF was carried out by “NMF” package and the optimal number of clusters is selected as the coexistence correlation coefficient [34]. The K–M survival analysis was performed to predict the diverse prognosis of ccRCC patients under this novel classification [35]. The proportion of main clinicopathological characteristics in each cluster was displayed as stacked histograms. PCA was subsequently conducted to show the result of DRGs clustering in different clusters. Finally, the gene set variation analysis (GSVA) method was utilized to analyze the differences of molecular functions and pathways enriched in different clusters [36]. |log_2_FC| >  0.1 and adjusted *P* < 0.05 were considered to be significant. The KEGG and ontology gene sets (c2.cp.kegg.v7.5.1.symbols.gmt, c5.go.bp.v7.5.1.symbols.gmt, c5.go.cc.v7.5.1.symbols.gmt, c5.go.mf.v7.5.1.symbols.gmt) were all obtained from the GSEA database (https://www.gsea-msigdb.org/gsea/index.jsp) [37].

### 2.5. Recognition of TIME and Immune Patterns According to Novel Classification

ESTIMATE could use the unique characteristics of cancer tissue transcription spectrum to infer tumor cells and normal cells with different infiltration [38]. Four indicators, including immune score, stromal score, ESTIMATE score, and tumor purity, were applied to identify TIME of each sample through “ESTIMATE” package. CIBERSORT, a deconvolution algorithm based on gene expression pattern, was employed to quantify the composition of cells involved in the immune response [39]. The abundance of infiltrating immune cells was measured to identify immune patterns patients in different clusters. The main immune checkpoints of ccRCC patients were summarized from relevant studies [1, 13, 40–43]. Moreover, differential analysis was conducted to reveal the different expression levels of immune checkpoints. The K–M analysis was performed to explore the association between immune patterns and patient survival. Different immunotherapeutic scores of ccRCC patients in different clusters were also calculated, and subsequently, the results were visualized through “ggplot2” package [44].

### 2.6. Construction and Validation of a Prognostic Risk Model Based on DRGs

The merged data consisting of TCGA-KIRC dataset and GSE29609 dataset were treated as the training cohort while the TCGA-KIRC dataset was treated as the validation cohort. First, WGCNA was utilized to identify modules related to the prognosis of ccRCC in the training cohort [45]. Subsequently, the univariate cox regression analysis was employed to select statistically significant DRGs (*P* < 0.01) and the prognostic value in the critical module was evaluated through “survival” package. Then, the LASSO regression analysis was carried out for further selection of prognosis-related DRGs and a risk model with these DRGs was constructed to predict the prognosis of ccRCC patients [46]. The risk score of each sample was calculated according to formula (1):

(1) $$\begin{array}{c} Risk\;score={\mathrm{Exp}}_{GENE1}\times \beta_{1}+{\mathrm{Exp}}_{GENE2}\times \beta_{2}+\ldots +{\mathrm{Exp}}_{GENEn}\times \beta_{n}. \end{array}$$

In which “Exp” stands for the expression levels of DRGs and “*β*” represents the regression coefficients of DRGs. Based on the median risk score, all patients could be divided into two types: low-risk and high-risk. The K–M survival analysis was performed to compare patient survival in the two risk groups. The concordance index and the ROC curve analysis were applied to evaluate the accuracy of this risk model [47]. In addition, the validation cohort was also used to further verify the performance of this prognostic risk model in predicting 1-year, 3-year, and 5-year survival rates.

### 2.7. Development and Evaluation of a Prognostic Nomogram

Univariate and multivariate cox regression analyses were performed in both the training cohort and the validation cohort to determine which were independent clinicopathological characteristics. Based on “rms” package, these independent characteristics and risk score were all used for the development of a prognostic nomogram for clinical practice [48]. Then, the accuracy of this nomogram was identified through calibration curves and discrimination performance was evaluated through C-index and ROC curves. Finally, this nomogram was validated in validation cohort.

### 2.8. Prediction of the Immunotherapeutic Response and Drug Sensitivity

TIDE (https://tide.dfci.harvard.edu/) is a computational method that simulated tumor immune escape by combining with the expression patterns of T cell dysfunction and rejection [49]. Based on the preprocessing data, the TIDE algorithm was carried out to predict the clinical response of immune checkpoint blocking (ICB) in ccRCC patients. Furthermore, “pRRophetic” package was employed to estimate the half maximum inhibitory concentration (IC50) values of compounds or inhibitors as a reference for clinical chemotherapy and targeted therapy of ccRCC patients in different risk groups or clusters [50].

## 3. Results

### 3.1. Identification of Cell Clusters Using scRNC-seq Data

**Figure 1:**
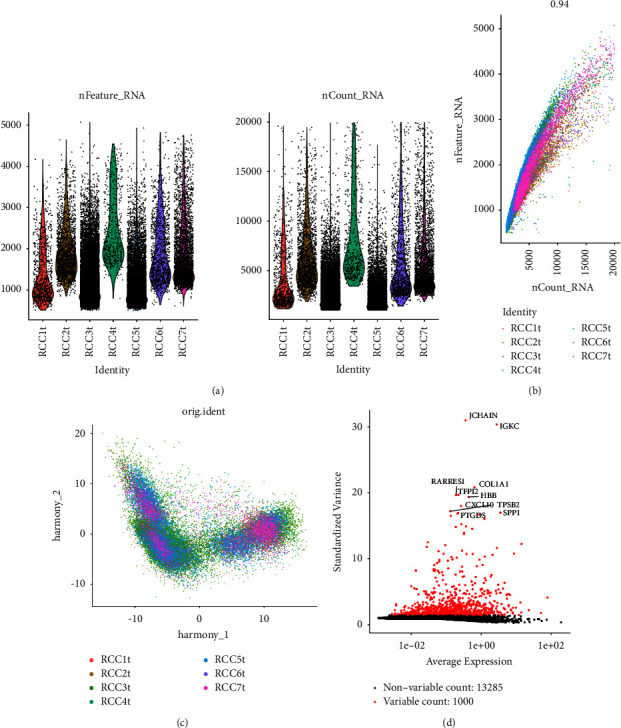
Preprocessing of the scRNA-seq data: (a) violin plots of the RNA information of processed scRNA-seq data, (b) scatter plot of the correlation between the numbers of detected genes and sequencing depth, (c) scatter plot of the batch effect after correction, and (d) scatter plot of 1,000 highly variable genes.

After the preprocessing of scRNA-seq data, including quality control, normalization, and batch effect correction, 32,400 cells from the GSE156632 dataset were included in the analysis (Figure 1(a)). The number of genes detected was significantly correlated with the sequencing depth (*R* = 0.94, Figure 1(b)). The dimensional reduction plot displayed the batch effect after correction (Figure 1(c)). Then, 14,285 genes were identified and top 1,000 genes were recognized as highly variable genes through variance analysis (Figure 1(d)). Available dimensions were determined through a principal component analysis (PCA), and subsequently, related genes were identified in each principal component (PC). The dot plots and heatmaps showed the expression levels of 30 significantly related genes in 6 top PCs (Figures S1a–S1c). Cell cluster analysis was performed on 11 PCs with a *P* value <0.05 (Figures S1c and S1d).

**Figure 2:**
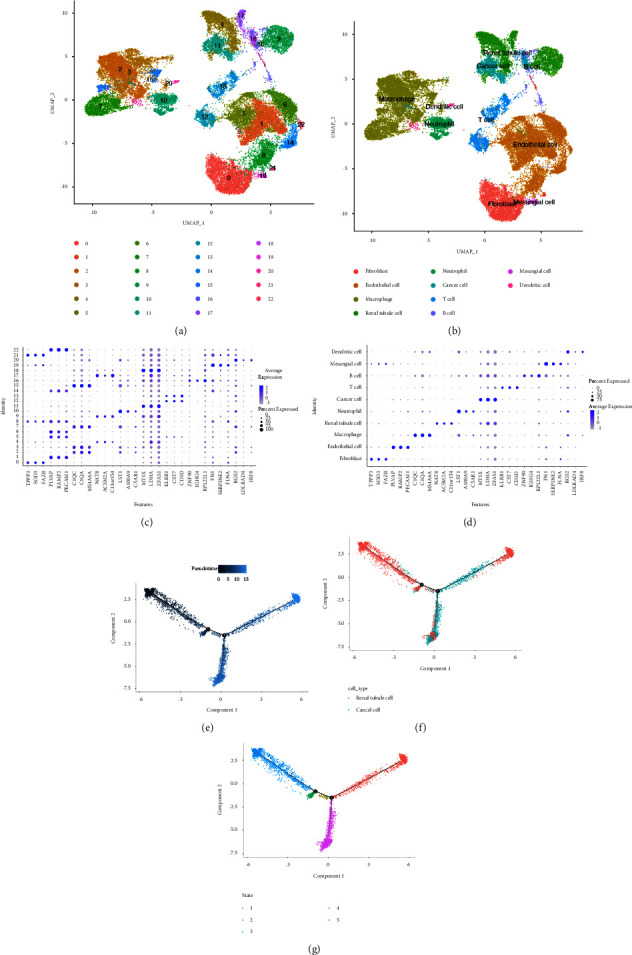
Cell clustering and trajectory analysis based on the scRNA-seq data: (a) scatter plot of 23 clusters processed by the UMAP algorithm, (b) scatter plot of 10 cell types obtained through annotation, (c–d) dot plot of the expression of major marker genes in different clusters and cell types, and (e–g) differentiation trajectory of 3 branches with diverse pseudotime, cell types, and states.

Afterward, the UMAP algorithm was applied to classify 32,400 cells into 23 clusters (Figure 2(a)). The top 5 differentially expressed marker genes of each cluster were visualized as a dot plot (Figure S2a). According to these marker genes, the cells distributed in 23 clusters were annotated (Figure 2(b)). The expression of major marker genes representing different cell types was visualized as dot plots (Figures 2(c) and 2(d)). As a result, clusters 0 and 21 with 4,297 cells were annotated as fibroblasts; clusters 1, 5, 6, 8, 14, and 22 with 10,015 cells were annotated as endothelial cells; clusters 4, 9, and 17 with 4,266 cells were annotated as renal tubule cells; cluster 19 with 185 cells was annotated as mesangial cells; clusters 11 and 18 with 1,642 cells were annotated as cancer cells; clusters 2, 3, 7, and 15 with 8,356 cells were annotated as macrophages; cluster 10 with 1,369 cells was annotated as neutrophils; clusters 12 and 13 with 1,721 cells were annotated as T cells; cluster 16 with 391 cells was annotated as B cells; and cluster 20 with 158 cells was annotated as dendritic cells.

### 3.2. Trajectory Analysis and DRGs Identification

Previous studies have shown that cancer cells are mostly differentiated from renal tubular epithelial cells in ccRCC. Trajectory analysis was conducted on renal tubule cells and cancer cells. We identified 3 branches with diverse differentiation statuses, termed branch I, II, and III. Most renal tubule cells were distributed in branch I (state 4) while cancers cells were mainly located in branches II and III (state 1 and 5). Therefore, branch I could be regarded as the root of differentiation trajectory and then differentiated into branches II and III. Interestingly, cancer cells in branch II, named type I cancer cells, were totally from cluster 11 and cancer cells in branch III, named type II cancer cells, were totally from cluster 18 (Figures 2(e)–2(g)). Based on the gene set enrichment analysis, we obtained distinct molecular mechanisms and pathways of two types of cancer cells (Figures S2b and S2c). In detail, type I cancer cells were related to the occurrence and development of cancer while type II cancer cells were involved in energy and material metabolism. Differential analysis was performed to identify pseudotime-dependent marker genes. Finally, a total of 715 marker genes were defined as DRGs and brought into the following analysis (Supplementary Table 4).

### 3.3. Classification for ccRCC Patients According to DRGs

**Figure 3:**
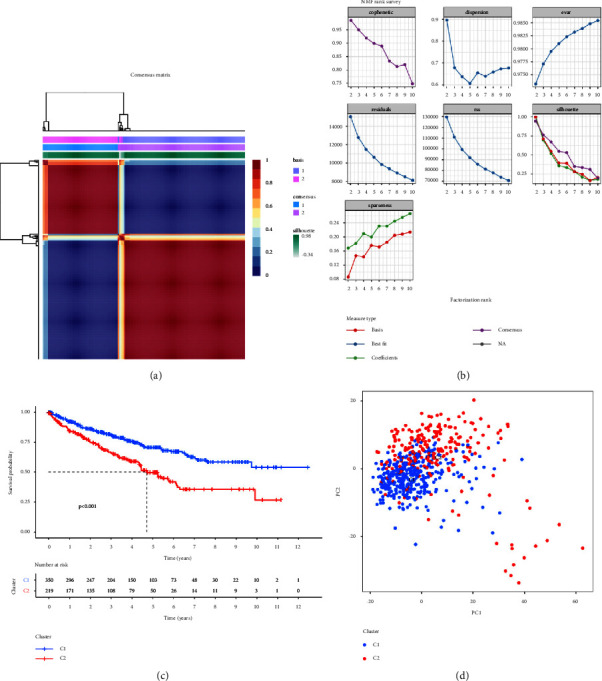
Classification constructed for ccRCC patients according to DRGs: (a–b) construction of classification through NMF algorithm, (c) Kaplan–Meier survival analysis for patients in 2 clusters, and (d) scatter plot of the classification of patients through PCA.

**Figure 4:**
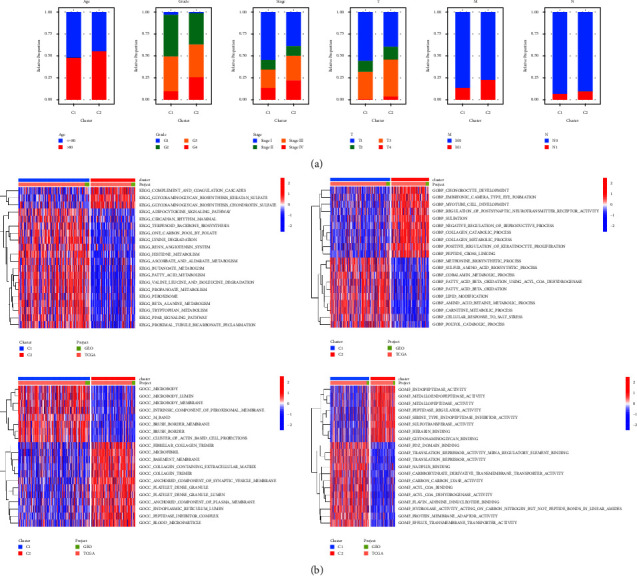
Comparisons of clinicopathological characteristics and differentially analysis of enriched functions between 2 clusters: (a) stacked histograms of the proportion of clinicopathological characteristics and (b) heatmaps of the diverse functional annotations.

All ccRCC patients were divided into 2 clusters with the coexistence correlation coefficient (*K* = 2) by the NMF clustering analysis (Figures 3(a) and 3(b)). K–M survival analysis showed that patients in cluster 2 had worse overall survival compared with patients in cluster 1 (Figure 3(c)). PCA demonstrated that this classification could distinguish ccRCC patients significantly (Figure 3(d)). Patients in cluster 2 had the clinicopathological features of higher levels of age, grade, and stage, which was consistent with the survival analysis (Figure 4(a)). Finally, differential analyses of biological process, molecular function, cellular component, and pathway were performed on 2 clusters and the results manifested that, different from cluster 1, ccRCC of cluster 2 was mainly related to immune responses and tumor mechanisms (Figure 4(b)). In general, the findings mentioned above showed that this novel classification of ccRCC patients based on DRGs was reliable and could be useful to distinguish survival outcomes of different populations in clinical practice.

### 3.4. Recognition of TIME and Immune Patterns According to Novel Classification

**Figure 5:**
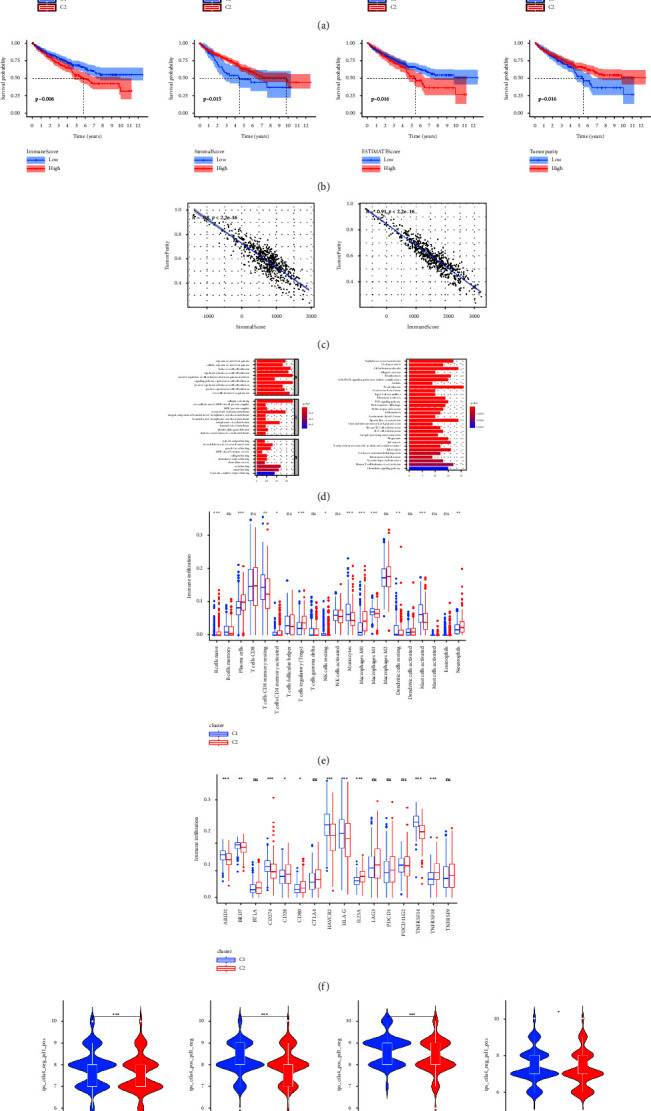
Identification of diverse TIME, immune gene pattern, and immunotherapeutic response in 2 clusters: (a) violin plots of 4 indicators of TIME between C1 and C2, including immune score, stromal score, ESTIMATE score, and tumor purity; (b) K–M survival analysis for high and low TIME score ccRCC patients; (c) scatter plots of the correlation between immune, stromal score and tumor purity; (d) bar plots of the results of functional enrichment analysis; (e) box plots of the abundances of different infiltrating immune cells; (f) box plots of the expression levels of immune checkpoints; and (g) violin plots of the immunotherapy scores.

ESTIMATE algorithm calculated the different abundance of immune and stromal cells and tumor purity in 2 clusters. Compared with ccRCC patients in cluster 1, ccRCC patients in cluster 2 had the higher immune, stromal, and ESTIMATE score and lower tumor purity (Figure 5(a)). The K–M survival analysis explored the correlation of TIME and overall survival in 2 clusters and the results indicated that ccRCC patients in cluster 1 tended to have a better prognosis (Figure 5(b)). Correlation analysis shows that both levels of infiltrating immune cells and stromal cells were negatively related to the level of tumor purity (Figure 5(c)). Based on the functional enrichment analysis of differentially expressed genes between different tumor purity levels, we found that the main GO and KEGG terms were all related immune reaction (Figure 5(d)). Moreover, the CIBERSORT algorithm was employed to make a further analysis of immune cell infiltration. From the analysis results, Naive B cells, plasma cells, CD4 memory resting T cells, regulatory T cells, M0 macrophages, and neutrophils were significantly more abundant in cluster 2 while CD4 memory activated T cells, resting NK cells, monocytes, M1 macrophages, resting dendritic cells, and resting mast cells were significantly more abundant in cluster 1 (Figure 5(e)). Patients with higher infiltration of memory B cells, M0 macrophages, M1 macrophages, activated NK cells, plasma cells, CD8 T cells, follicular helper T cells, and regulatory T cells got worse overall survival while patients with higher infiltration of activated dendritic cells, resting dendritic cells, eosinophils, M2 macrophages, resting mast cells, monocytes, and CD4 memory resting T cells got better overall survival (Figure S3). According to the analysis of immune checkpoints, the expression levels of CD28, CD80, IL23A, and TNRSF18 were higher in cluster 2 patients (Figure 5(f)). Patients with lower expression levels of CD80, CTLA4, IL23A, LAG3, PDCD1, TNFRSF9, TNFRSF14, and TNFRSF18 or higher expression levels of ARID2, BRD7, BTLA, CD274, HAVCR2, HLA-G, and PDCD1LG2 tended to have a better overall survival rate (Figure S4). Finally, the different effects of anti-PD1 and anti-CTLA4 immunotherapies were estimated across 2 clusters (Figure 5(g)). The scores of each type immunotherapy in cluster 1 were significantly higher than those in cluster 2 and it indicated that cluster 1 patients were more likely to benefit from immunotherapy.

### 3.5. Construction and Validation of DRGs Based on a Prognostic Risk Model

**Figure 6:**
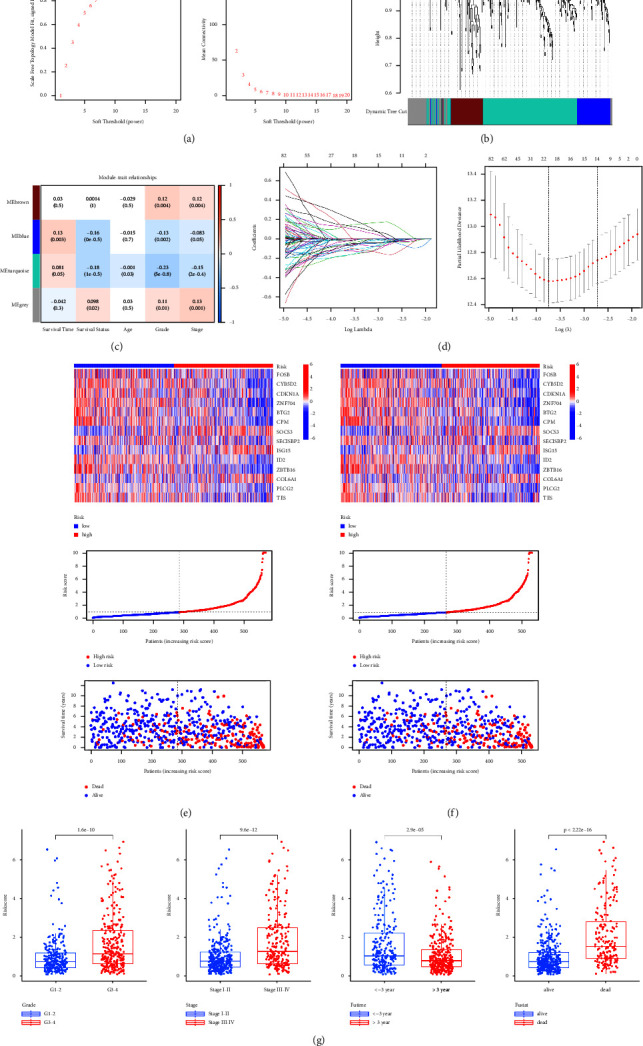
Construction and validation of the prognostic risk model based on DRGs: (a) nature of the network topology constructed with unique power values and the relationship between power values and average connectivity, (b) discrete modules of obtained through DRGs clustering, (c) module diagram of the correlation between clinicopathological characteristics and identified modules, (d) coefficient profile plot of the log (lambda) sequence of the LASSO model, (e–f) heatmaps of the expression levels of prognostic genes, curves of the risk score, and scatter plots of survival status in the training and validation cohort, and (g) box plots of the correlation between clinical variables and risk score.

**Figure 7:**
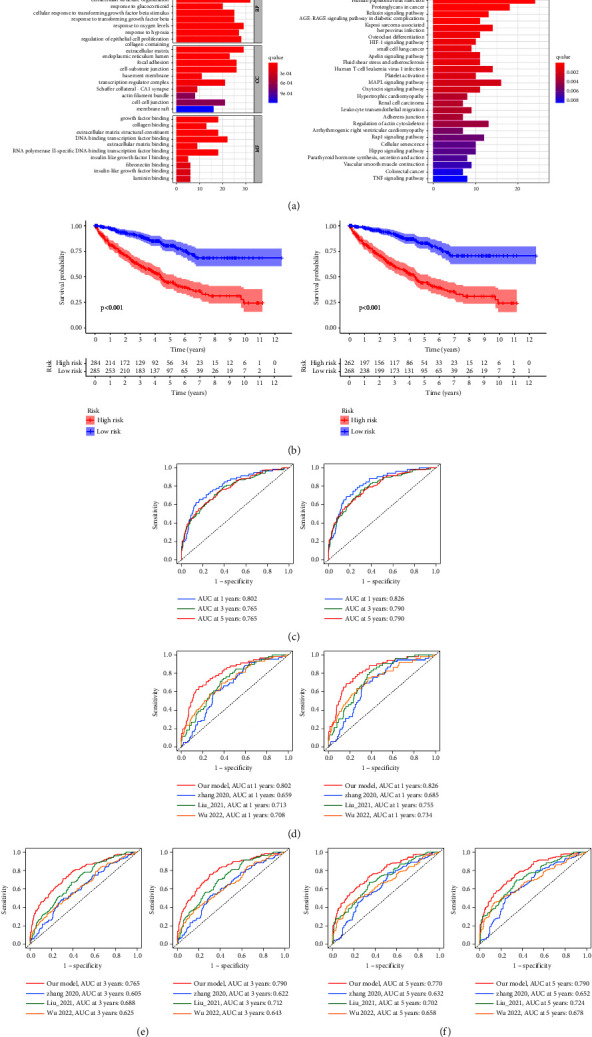
Depth analysis according to the prognostic risk model: (a) bar plots showing the results of functional enrichment analysis based on DRGs, (b) K–M survival analysis of different risk patients in training and validation cohort, (c) ROC curves of the predictive efficiency of our model in training and validation cohort, and (d–f) ROC curves of the diverse predictive efficiency between our model and other published models in training and validation cohorts.

The WGCNA algorithm was carried out to determine modules related to prognosis of ccRCC (Figures 6(a) and 6(b)). Based on the average linkage hierarchical clustering and soft threshold power, 4 modules were identified and the turquoise module was significantly associated with all clinicopathological characteristics of ccRCC patients (Figure 6(c)). Subsequently, the univariate cox analysis was employed to screen out all DRGs with prognostic values in the turquoise module. The result of the univariate cox analysis is listed in Supplementary Table 5. Finally, a prognostic risk model with 14 DRGs, including BTG2, CDKN1A, COL6A1, CPM, CYB5D2, FOSB, ID2, ISG15, PLCG2, SECISBP2, SOCS3, TES, ZBTB16, and ZNF704, was established using the LASSO regression algorithm (Figure 6(d)). A total of 284 patients were included in the high-risk group and the rest were included in the low-risk group. These 14 DRGs with corresponding coefficients are listed in Supplementary Table 6. The expression levels of 14 DRGs were diverse among different cell types (Figure S5a). The risk scores of patients could be calculated according to this model. Therefore, taking the median risk score as the threshold, all patients can be divided into the two risk groups. The association among data source, classification, risk score, and survival status is shown as a Sankey diagram (Figure S5b). Patients in the cluster 2 had significantly higher risk scores than those in the cluster 1 (Figure S5c). The expression levels of SOCS3, ISG15, and COL6A1 were proportionate to the risk scores. It indicated these 3 DRGs may act as risk genes. On the contrary, other DRGs were regarded as protective genes. The risk score had a negative correlation with the survival time and survival status of patients (Figures 6(e) and 6(f)). It was clear that the risk score statistically correlated with grade, stage, survival time, and status (Figure 6(g)). Moreover, the expression levels of these 14 DRGs were significantly different between patients of the two risk groups (Figure S6a). Enrichment analysis indicated functional significance of DRGs in ccRCC. (Figure 7(a)). The K–M survival analysis demonstrated that patients with high-risk scores had a worse overall survival rate than those with a low-risk score either in the training or validation cohort (Figure 7(b)). Receiver operating characteristic (ROC) analysis manifested that this model showed excellent performance in predicting overall survival rate of ccRCC patients. The areas under the ROC curves (AUC) to predict 1-year, 3-year, and 5-year overall survival were 0.802, 0.765, and 0.765 in training cohort, and 0.826, 0.790, and 0.790 in validation cohort (Figure 7(c)), respectively. The effect of grade or stage as a subvariable to predict overall survival was also better (Figure S6b). Comparing published prognostic risk models with our model, the accuracy of our model was proved to be better than others. In detail, the AUC value of the best model to predict 1-year, 3-year, and 5-year overall survival was 0.713, 0.688, and 0.702 in training cohort and 0.755, 0.712, and 0.724 in validation cohort (Figures 7(d)–7(f)), respectively.

### 3.6. Development and Validation of a Prognostic Nomogram

**Figure 8:**
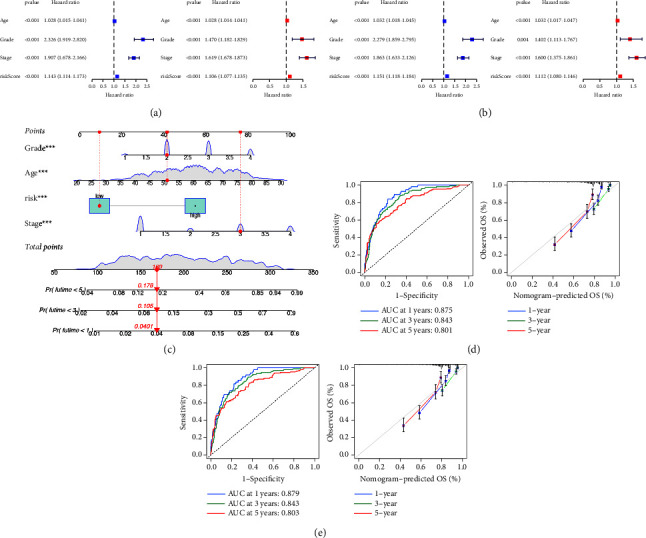
Development and validation of prognostic nomogram: (a–b) forest plots of the univariate and multivariate cox regression in training and validation cohorts, (b) nomogram composed of age, grade, stage, and risk score with the prediction of overall survival rate, (d–e) ROC and calibration curves of nomogram for the prediction of overall survival rate in training and validation cohorts.

In the training cohort, the univariate cox analysis showed that age, grade, stage, and risk score all had a prognostic value. The multivariate cox analysis indicated that all variables can be independent features to predict the prognosis of ccRCC patients (Figure 8(a)). Subsequently, the same results were obtained from the validation cohort (Figure 8(b)). Then, a prognostic nomogram integrating age, grade, stage, and risk score was developed to offer a clinically applicable method for the prediction of individual prognosis (Figure 8(c)). The ROC curves showed an excellent ability of this model for the prediction of 1-year, 3-year, and 5-year overall survival rates in the training cohort and the AUC values were 0.875, 0.843, and 0.801, respectively. The calibration curves for predicting 1-year, 3-year, and 5-year overall survival were also close to the actual observations (Figure 8(d)). Of course, the same analysis was conducted in the validation cohort (Figure 8(e)).

### 3.7. Prediction of the Immunotherapeutic Response and Drug Sensitivity

**Figure 9:**
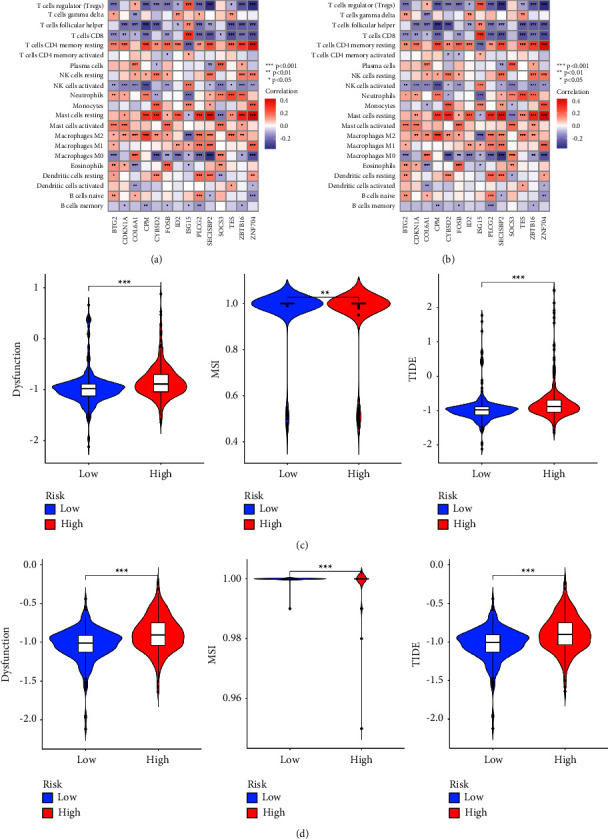
Prediction of immunotherapeutic response and drug sensitivity: (a–b) heatmaps of correlation between the expression levels of 14 DGRs and the abundance of infiltrating immune cells in training and validation cohorts and (c–d) violin plots of immunotherapeutic response in different risk groups based on training and validation cohorts.

Correlation analysis manifested the expression of 14 model DRGs was significantly correlated with the abundance of infiltrating immune cells in both the training and validation cohorts. In particular, ISG15 had a different TIME pattern from other DRGs. (Figures 9(a) and 9(b)). Compared with ccRCC patients with a low-risk score, patients with high-risk score tended to have a better respond to immunotherapy. The similar results were also obtained from the validation cohort (Figures 9(c) and 9(d)). Furthermore, the response of 83 compounds or inhibitors was significantly different in the two risk groups, in which 31 compounds or inhibitors had a better drug response in the high-risk group while 52 compounds or inhibitors had a better drug response in the low-risk group (Supplementary Table 7). Meanwhile, a total of 70 compounds or inhibitors were significantly different in 2 clusters, in which 41 compounds or inhibitors had a better drug response in cluster 1 (Supplementary Table 8).

## 4. Discussion

ccRCC is the most common and fatal renal system tumor with high levels of intratumor heterogeneity [51, 52]. In recent years, intratumor heterogeneity is regarded as one of the potential causes of therapeutic drug resistance [53]. Therefore, it is necessary to explore cellular heterogeneity in ccRCC samples using the scRNA-seq analysis. So far, the study on the differentiation of ccRCC cells is still very limited and it is also unclear whether the differentiation status of ccRCC cells is associated with the prognosis and therapy response [1, 54]. In this study, we employed the scRNA-seq data in GEO database to reveal the differentiation status of ccRCC cells. Based on novel classification, ccRCC patients could be divided into 2 clusters with diverse clinicopathological characteristics. At the same time, TIME, immune gene expression, and immunotherapeutic response represented significant difference in 2 clusters. Subsequently, a risk model composed of 14 prognostic DRGs was established to predict the prognosis of ccRCC patients and a nomogram model integrating clinicopathological characteristics and risk score was constructed for clinical practice. Finally, we compared the immunotherapeutic response and drug sensitivity of ccRCC patients in the two risk groups to explore the possibility of clinical therapy.

Intratumor heterogeneity is characterized by cells with different features in a single tumor. These cells show different cell collections with different molecular characteristics or differentiation status [55]. A total of 23 cell clusters were identified and subsequently 10 cell types were obtained through annotation. In view of the fact that most cancer cells were considered to be derived from renal tubular epithelial cells, we chose renal tubular cells and cancer cells for differentiation trajectory analysis. The differentiation trajectory showed that renal tubular cells was the root of differentiation and then differentiated into 2 diverse branches representing 2 different types of cancer cells. According to the expression patterns of DRGs, a novel classification with different clinicopathological characteristics was performed on ccRCC patients. The association between classification and differentiation status indicated that the prognosis and immunotherapy response were related to the cell differentiation status. The study of DRGs recognition could be helpful to better understand the occurrence and development of ccRCC. Numerous studies have showed that cellular signaling pathways and transcriptional cascades involved in differentiation process were associated with the occurrence and development of malignant tumors [56–58]. Differentiation therapy provided a new idea for the therapy of malignant tumors which induced cancer cells by transforming signal events and then guided them to a status of higher differentiation and lower malignancy [59, 60]. Although great progress has been made in the differentiation therapy in ccRCC, the specific molecular mechanism and therapy targets needed to be further studied [61, 62]. In this study, we identified prognosis-related DRGs to provide more reference for clinical therapy. So far, few studies have focused on the correlation between differentiation status and TIME in ccRCC. In our study, patients in cluster 2 tended to have a higher level of infiltrating immune cells and lower level of tumor purity compared with patients in cluster 1. Moreover, patients in cluster 2 were sensitive to immunotherapy and it was consistent with the result that patients with high risk tended to have a better immunotherapeutic response. From the published studies, we found that 5 model DRGs were proved to be associated with ccRCC. In detail, Sima et al. have investigated the impact of BTG2 on growth, migration, and invasion of ccRCC cells and found overexpressed BTG2 could inhibit proliferation, migration, and invasion of ccRCC cells [63]. PANDAR, promoter of CDKN1A antisense DNA damage activated RNA, had significantly upregulated expression in tumor tissues and could serve as an independent predictor of overall survival in ccRCC [64]. Moreover, Zhu et al. studied the therapeutic potential of LSD1 inhibitors in ccRCC treatment and discovered that inhibition of LSD1 could decrease the H3K4 demethylation at the CDKN1A gene promoter and it was associated with P21 upregulation and cell cycle arrest at G1/S in ccRCC cells [65]. The COL6A1 was a gene encoding the alpha 1 polypeptide subunit of collagen 6 and ccRCC patients were discovered to have significantly higher COL6A1 scores and intensities [66]. Like us, Wan et al. included ISG15 as one of the prognostic predictors in a constructed risk model of ccRCC [67]. Urbschat et al. observed significantly lower SOCS3 messenger RNA levels in tumor tissues compared to healthy tissues and concluded SOCS3, as a negative regulator, participated in regulation of ccRCC together with STAT3 [68]. At present, it was the first time to find other model DRGs, including CPM, CYB5D2, FOSB, ID2, PLCG2, SECISBP2, TES, and ZBTB16, were related to ccRCC, which needed further study. To sum up, all findings emphasized the possibility to predict TIME and immunotherapeutic response of ccRCC patients based on prognostic DRGs.

Compared to the AUC value of other prognosis risk models, our model showed a higher accuracy to predict the prognosis of ccRCC patients. The TIDE analysis showed that patients with high risk responded better to immunotherapy than patients with low risk. It indicated that risk score could also be applied as an indicator for the prediction of immunotherapeutic response. In addition, we found that the response of 83 compounds or inhibitors was significantly different in the two risk groups which could be used as a reference for clinical therapy. We focused on 32 compounds or inhibitors showing better response in the high-risk group. Fortunately, several results of compounds or inhibitors were consistent with published studies. For example, in the presence of AKT inhibitor VIII, a pan-AKT inhibitor, ART reduced more ccRCC cell proliferation, migration, and invasion than in the absence of AKT inhibitor VIII [69]. AZD6482 selectively inhibited migration, invasiveness, and colony formation of ccRCC cells with SETD2 mutations [70]. In the xenotransplantation model of mice, AZD8055 achieved significantly better tumor growth inhibition and prolonged survival time of mice than sirolimus or excipients [71]. Gao et al. have provided evidence to elucidate that miR-200c could sensitize ccRCC cells to sorafenib or imatinib to inhibit cell proliferation, at least partly by targeting HO-1 [72]. von Hippel-Lindau (VHL) gene mutation was the driving force of various forms of ccRCC and MG-132 mediated proteasome inhibition could make VHL wild type cells sensitive to zafirlukast-induced cell death [73]. The synergistic effect of sAXL with pazopanib and axitinib could reduce the growth of xenograft derived from ccRCC patients, which supported the combination of AXL inhibitors and antiangiogenic agents in the treatment of ccRCC [74]. Thapsigargin had the highest performance in the treatment of early metastatic ccRCC and could be used as an effective small molecule drug to treat early metastatic ccRCC [75]. Meanwhile, the role of other compounds or inhibitors in ccRCC needed for further confirmation.

The current study had some drawbacks. First of all, all data were obtained from the published database rather than our own dataset. Thus, the detailed clinical information was incomplete and could not be included in the nomogram model. Given that the lack of available data, more validation should be performed on other ccRCC cohorts. On the other hand, the specific mechanism of most DRGs was not clear in ccRCC and our study needed to be further verified through cellular biological experiments.

## 5. Conclusion

This study highlighted the cell differentiation trajectory of ccRCC cells and manifested a potential impact on the prediction of the prognosis and immunotherapeutic response in ccRCC patients. In detail, a novel classification of ccRCC patients was constructed and proved to be reliable in the prediction of diverse prognosis, TIME pattern, and immunotherapeutic response. Cell differentiation-related genes were identified; then, a prognostic risk model with these genes was constructed to predict the prognosis and immunotherapeutic response of ccRCC patients with different risk scores. We also established a nomogram composed of clinicopathological characteristics and risk score for diagnosis and estimated the drug sensitivity of ccRCC patients with different risk scores for treatment.

### 5.1. Statistical Analysis Methods

The comparison in multiple groups was performed using the Kruskal–Wallis test and the comparison between the two groups was based on Wilcoxon test. The Pearson correlation test was used to study the correlation between normally distributed variables while the correlation between nonnormally distributed variables was evaluated by using the Spearman correlation test. The Chi square test was used to analyze the distribution of categorical variables among subgroups, and the Student's *t*-test was used to compare continuous data between the two subgroups. In the K–M analysis, the log rank test was carried out to examine statistical difference. All data analysis and visualization were completed using R version 4.0.3.

## Data Availability

All datasets included in the study are available in the TCGA dataset (https://portal.gdc.cancer.gov/) and GEO database (https://www.ncbi.nlm.nih.gov/geo/query/acc.cgi?acc=GSE29609, https://www.ncbi.nlm.nih.gov/geo/query/acc.cgi?acc=GSE156632).

## Abbreviations

RCC: Clear cell renal cell carcinoma
scRNA-seq: Single-cell RNA sequencing
DRGs: Differentiation-related genes
TIME: Tumor-immune microenvironment
TCGA: The Cancer Genome Atlas
GEO: The Gene Expression Omnibus
PCA: Principal component analysis
UMAP: Uniform manifold approximation and projection
TPM: Transcripts per million
NMF: Nonnegative matrix factorization
GSVA: Gene set variation analysis
WGCNA: Weighted gene co-expression network analysis
LASSO: Least absolute shrinkage and selection operator
ROC: Receiver operating characteristic
AUC: Area under curve
IC50: Half maximum inhibitory concentration.

## Supplementary Materials

Supplementary Materials Figure S1: PCA analysis based on scRNA-seq data: (a-b) dot plots and heatmaps of 30 main genes in top 6 PCs, (c-d) identification of PCs with significant difference. Figure S2: Clustering and differentiation trajectory analysis based on the scRNA-seq data: (a) dot plot of the top 5 marker genes in 23 clusters, (b-c) functional annotation for 2 branches with different types of cancer cells. Figure S3: The K-M survival analysis for patients with diverse abundance of infiltrating immune cells (*P* < 0.05). Figure S4: The K-M survival analysis for patients with diverse expression levels of immune checkpoints (*P* value <0.05). Figure S5: The depth analysis according to the prognostic risk model: (a) scatter plots of the 14 DRGs among different cell types, (b) Sankey diagram of the associations among classification, risk score, survival status, and data source, (c) violin plot of risk score between 2 cluster. Figure S6: The depth analysis of 14 DRGs expression patterns and clinicopathological variables: (a) box plots of the expression levels of 14 DRGs in different risk groups in both the training and validation cohort, (b) the ROC curves of the predictive ability of clinicopathological characteristics in both the training and validation cohort. Supplementary Table 1. Clinicopathological features of samples in TCGA-KIRC. Supplementary Table 2. Clinicopathological features of samples in GSE29609. Supplementary Table 3. Clinicopathological features of samples in GSE156632. Supplementary Table 4. The marker genes related to cell differentiation. Supplementary Table 5. Prognostic DRGs identified by the univariate cox analysis. Supplementary Table 6. DRGs with corresponding coefficients used in our model. Supplementary Table 7. Two types of compounds or inhibitors with distinct drug response in the two risk groups. Supplementary Table 8. Two types of compounds or inhibitors with distinct drug response in two clusters.
