# Supplementary material for: Cell Differentiation Trajectory Predicts Prognosis and Immunotherapeutic Response in Clear Cell Renal Cell Carcinoma
Source: Genet Res (Camb). 2022 Nov 29;2022:8422339. doi: 10.1155/2022/8422339 (PMC9726251; doi:10.1155/2022/8422339)
Supplement: Supplementary Materials — Figure S1: PCA analysis based on scRNA-seq data: (a-b) dot plots and heatmaps of 30 main genes in top 6 PCs, (c-d) identification of PCs with significant difference. Figure S2: Clustering and differentiation trajectory analysis based on the scRNA-seq data: (a) dot plot of the top 5 marker genes in 23 clusters, (b-c) functional annotation for 2 branches with different types of cancer cells. Figure S3: The K-M survival analysis for patients with diverse abundance of infiltrating immune cells (P < 0.05). Figure S4: The K-M survival analysis for patients with diverse expression levels of immune checkpoints (P value <0.05). Figure S5: The depth analysis according to the prognostic risk model: (a) scatter plots of the 14 DRGs among different cell types, (b) Sankey diagram of the associations among classification, risk score, survival status, and data source, (c) violin plot of risk score between 2 cluster. Figure S6: The depth analysis of 14 DRGs expression patterns and clinicopathological variables: (a) box plots of the expression levels of 14 DRGs in different risk groups in both the training and validation cohort, (b) the ROC curves of the predictive ability of clinicopathological characteristics in both the training and validation cohort. Supplementary Table 1. Clinicopathological features of samples in TCGA-KIRC. Supplementary Table 2. Clinicopathological features of samples in GSE29609. Supplementary Table 3. Clinicopathological features of samples in GSE156632. Supplementary Table 4. The marker genes related to cell differentiation. Supplementary Table 5. Prognostic DRGs identified by the univariate cox analysis. Supplementary Table 6. DRGs with corresponding coefficients used in our model. Supplementary Table 7. Two types of compounds or inhibitors with distinct drug response in the two risk groups. Supplementary Table 8. Two types of compounds or inhibitors with distinct drug response in two clusters. [file 8422339.f1.zip › Supplementary table 8.docx]

**Supplementary table 8. Two types of compounds or inhibitors with distinct drug response in two clusters**

| **Cluster** | **Name** |
| --- | --- |
| **C1** | A.443654, A.770041, AG.014699, AICAR, AMG.706,  AZD7762, BI.2536, BI.D1870, BMS.509744, BMS.536924,  Bortezomib, Bryostatin.1, BX.795, Camptothecin, CCT018159,  CEP.701, CGP.60474, CGP.082996, CI.1040, CMK, Dasatinib,  DMOG, Docetaxel, Elesclomol, GNF.2, GSK269962A,  GW843682X, JNJ.26854165, JNK.Inhibitor.VIII, JW.7.52.1,  KIN001.135, KU.55933, LFM.A13, NU.7441, Nutlin.3a,  NVP.BEZ235, NVP.TAE684, Paclitaxel, Parthenolide,  RDEA119, RO.3306 |
| **C2** | ABT.263, AKT.inhibitor.VIII, AS601245, ATRA, AZD8055,  BAY.61.3606, Bleomycin, CHIR.99021, EHT.1864, Embelin,  Epothilone.B, Erlotinib, Etoposide, FH535, Gemcitabine,  GSK.650394, GW.441756, IPA.3, JNK.9L, Metformin,  Methotrexate, Mitomycin.C, MK.2206, NSC.87877,  Obatoclax.Mesylate, OSI.906, PAC.1, Pyrimethamine, QS11 |
